# Breastfeeding and Early Infant Feeding Practices Among Women in the Hunter New England Region of New South Wales, Australia: A Cross Sectional Study

**DOI:** 10.1002/hpja.70028

**Published:** 2025-03-17

**Authors:** Tessa Delaney, Jacklyn Jackson, Nayerra Hudson, Christophe Lecathelinais, Alison L. Brown, Sarah Young, Luke Wolfenden, Paul Craven, Margaret Hayes, Sinead Redman, John Wiggers, Jessica Pinfold, Rebecca Liackman, Daniel Groombridge, Nicole Nathan, Rachel Sutherland

**Affiliations:** ^1^ Hunter New England Local Health District Newcastle Australia; ^2^ School of Medicine and Public Health The University of Newcastle Callaghan Australia; ^3^ Hunter Medical Research Institute Newcastle Australia; ^4^ Northern Sydney Local Health District St Leonards Australia

**Keywords:** breastfeeding, complementary foods, infant feeding practices, infants, solid feeding, survey

## Abstract

**Issue Addressed:**

Exclusive breastfeeding to 6 months of age is recommended. Currently, there is a lack of Australian data exploring infant feeding behaviours and the sources of information women use to guide infant feeding decisions. This study aimed to describe (i) infant feeding practices (breastfeeding, infant formula/other fluids, introduction of solids) of women with infants aged 6–8 months; and (ii) the information sources women use most frequently and find most helpful to make decisions regarding infant feeding practices.

**Methods:**

Between August and October 2021, 356 mother‐infant dyads in the Hunter New England region of New South Wales, Australia, were surveyed. Descriptive statistics and Kaplan‐Meier survival analyses were used to describe infant feeding practices and their timing (age in months).

**Results:**

While breastfeeding was initiated in 97% of infants, only 1% were exclusively breastfed to 6 months of age. In the first month of life, 21% of infants received formula, increasing to 51% by 6 months. The mean age of introducing solids was 5.3 months. The most frequently used and helpful sources of information for infant feeding included friends and family, child and family health nurses, and digital sources (e.g., websites).

**Conclusions:**

Infant feeding practices reported by Australian mothers remain inconsistent with the recommendations and should remain a key focus of public health nutrition efforts.

**So What?:**

Opportunity exists to provide consistent, credible, and evidence‐based information via various modalities for both families and their support networks to promote best practice infant feeding.

## 1 Introduction

Breast milk contains the essential nutrition required for healthy infant growth and development in the first 6 months of life [1, 2]. Breastfeeding represents one of the most important modifiable health behaviours for early life and is shown to have major impacts on early morbidity and mortality [3]. Breastfeeding also promotes long‐term health benefits for children, having protective effects against overweight, obesity and decreased risk of developing chronic diseases such as type 2 diabetes later in life [1, 4, 5, 6, 7]. As such, international and national infant feeding guidelines [1, 2], including the 2013 Infant Feeding Guidelines published by the Australian National Health and Medical Research Council [2], recommend that infants are exclusively breastfed until around 6 months of age. At around 6 months, it is recommended that iron‐rich nutrient‐dense foods are introduced to complement breast milk, with the continuation of breastfeeding to 12 months and beyond [2]. However, the measurement of breastfeeding and associated infant feeding behaviours represents a complex matter, due to the interplay between breastfeeding and other infant feeding behaviours, including the use of infant formula, the introduction of other fluids (e.g., cow's milk, juice and cordials) and complementary soft/semi‐solid/solid foods. Research indicates that the introduction of infant formula and early introduction to other fluids and complementary foods are associated with rapid weight gain during infancy and are associated with an increased future risk of overweight, obesity, and other chronic diseases later in life [8, 9]. As such, the protection/promotion of breastfeeding and other infant feeding behaviours aligned with recommendations remains a key public health priority internationally.

Australia's most comprehensive national survey of infant feeding practices, the Australian National Infant Feeding Survey (ANIFS) was last undertaken in 2010–2011 [10]. The survey found that while most infants had initiated breastfeeding (96%) only 27% were exclusively breastfed to 4 months, and by 6 months this was as low as 2% [10]. While breastfeeding initiation rates are high, by 1 month 34% of infants had been introduced to formula, and by 2 and 6 months this had increased to 45% and 69%, respectively [10]. Additionally, 35% of infants aged 4 months had consumed soft/semi‐solid/solid foods, increasing to 92% by 6 months [10]. Despite the ANIFS offering a comprehensive understanding of infant feeding patterns in Australia, the report did not include data related to the early introduction of other fluids. Further, the study was undertaken over 10 years ago, and given the rapidly changing social and economic landscape, it may no longer represent current feeding practices [11].

A more recent cross‐sectional study undertaken by Reynolds and colleagues [12] surveyed 536 Australian women in the 5 months following birth. This study found approximately 93% of women had initiated breastfeeding; however, of the women who had ceased breastfeeding (*n* = 121), 51% (*n* = 62) breastfed for less than a month [12]. Of the 73% (*n* = 390) of women still breastfeeding at the time of the survey, 57% (*n* = 304) were exclusively breastfeeding, 13% (*n* = 68) were breastfeeding and formula feeding, and 3% (*n* = 18) were breastfeeding and commenced solids [12]. The Australian Feeding Infants and Toddler Study (OzFITS) 2021 also recently explored infant and toddler feeding practices within a national sample of caregivers up to 24 months post‐partum. This study found that by 4 months, only 39% of mothers exclusively breastfed, 51% had introduced breastmilk substitutes, and 25% had introduced solids. While this data reinforces the notion that more must be done to support breastfeeding and infant feeding behaviours in Australia, identifying the sources of information women access to inform their infant feeding decisions will be helpful for directing public health nutrition efforts.

For Australian mothers, one of the main barriers influencing the appropriate timing of solid introduction has been confusion around the recommended guidelines [13]. A recent (2019) qualitative analysis of Australian mothers identified that the main sources of information influencing infant feeding were the mother's own mother, friends and other mothers [14]. It also identified that mothers like to do their own research when considering the introduction of solid foods; however, the exact sources of information favoured by mothers were not identified [14]. As infant feeding decisions (i.e., early introduction to solids) can have significant short‐term and long‐term health implications [9], a more robust understanding of how mothers access sources of infant feeding information using quantitative methods is vital for informing programmes and policies that aim to promote evidence‐based infant feeding practices.

To our knowledge there are no published studies that have comprehensively described breastfeeding and associated early infant feeding practices, as well as explored how mothers (6–8 months post‐partum) access information to help inform their infant feeding practices, during a time they are likely experiencing a major transitional phase in infant feeding (i.e., introduction to solids). Exploring this information together will be useful for designing future population based infant feeding initiatives, and not only inform the best places to direct information, but also inform the best timing to provide this information. Therefore, to address this evidence gap, the current study aimed to (i) explore the breastfeeding and infant feeding practices (including introduction to solids, introduction to ‘other fluids’ and formula feeding) of women with infants aged 6 to 8 months in the Hunter New England (HNE) region of New South Wales and (ii) identify frequent sources of information women access to make helpful decisions around their infant feeding behaviours.

## 2 Materials and Methods

### 2.1 Ethics

The study is reported in accordance with the Strengthening the Reporting of Observational studies in Epidemiology (STROBE) guidelines [15]. Ethical approval to undertake the study was obtained from the HNE Human Research Ethics Committee (16/11/16/4.07), Aboriginal Health and Medical Research Council (1236/16) and the University of Newcastle Human Research Ethics Committee (H‐2017‐0032).

### 2.2 Study Design and Setting

A cross‐sectional survey was undertaken from August 2021 to October 2021 in the HNE Local Health District of New South Wales, Australia. The HNE Region covers 131 785 km^2^ and has a population of 920 370 people [16]. In 2020, there were 10,242 births within the HNE Local Health District, accounting for approximately 10% of births in NSW [17]. The survey was conducted via computer‐assisted telephone interviews (CATI) and collected data to examine current infant feeding practices in women 6–8 months post‐partum.

### 2.3 Participants

Similar to methods described by Reynolds et al. [12], women who had participated in a previous survey during their pregnancy and agreed to be contacted for future research were invited to participate in the study. Women were eligible for inclusion in the study if they:

- were ≥ 18 years of age
- had an infant 6–8 months of age
- were able to complete the survey unaided (i.e., had English language proficiency)

### 2.4 Recruitment

All eligible women (*N* = 713) received a written information statement inviting study participation, which was mailed to their postal address. The information statement outlined the purpose of the study, the method of data collection, and included an opt‐out telephone number for participants to contact should they choose to opt out of the study prior to the initial contact. The sample of eligible women was generated by combining electronic medical record data (i.e., child date of birth, live birth) and previous survey data (i.e., from those that had consented to be contacted for future research). A weekly sample of up to 100 women was approached to participate in the study over an 8‐week period. To maximise the sample frame, women with older infants (i.e., 8 months) were approached first to minimise ineligibility. Based on advice received from Aboriginal stakeholders, women were invited to take part in the CATI in two groups, as follows:

#### 2.4.1 Non‐Aboriginal and/or Non‐Torres Strait Islander Women

One week after information statements were mailed, non‐Aboriginal women were contacted via telephone and invited by a female interviewer to participate in the CATI. As per ethics approval, verbal consent to participate in the study was obtained from the women at the time of the call. A maximum of 10 phone calls were attempted over a two‐week period to invite study participation.

#### 2.4.2 Aboriginal and/or Torres Strait Islander Women and/or Women Who Access Aboriginal Maternal Health Services (AMIHS)

Women who identified as Aboriginal and/or Torres Strait Islander (via electronic health data) were sent a text message after the information statement was mailed, inviting them to participate and nominate their preferred method of participation as follows: option 1—phone survey; option 2—online survey; option 3—opt out. Women who opted to complete the survey online were provided with an individual survey link to their mobile number, which was active for 2 weeks. Women who opted to complete the survey via CATI and those who did not respond to the text message were contacted via telephone and invited to participate as per procedures described above. All women who opted to complete either the CATI or online survey were asked if they identified as Aboriginal or Torres Strait Islander or both (regardless of what had been previously recorded in health records). As per ethics and local consultation processes, women who identified as Aboriginal and/or Torres Strait Islander during the CATI were offered the choice of undertaking the survey with a female interviewer who identified as Aboriginal.

### 2.5 Data Collection Procedures

The CATI was administered over a 10‐week period between August and October 2021. CATI consent and survey data were stored in REDCap [18], an encrypted web application that stores and manages health service databases.

### 2.6 Outcomes & Measures

#### 2.6.1 Maternal and Infant Characteristics

The survey included items assessing the child's date of birth, child's weight at birth, whether the mother identifies as Aboriginal and/or Torres Strait Islander, residential postcode, highest level of education, and age of the child in months when the mother returned to work. Participant age was obtained from their previous antenatal survey.

#### 2.6.2 Knowledge of Recommendations for Exclusive Breastfeeding

Women were asked to identify the recommended age at which infants should be exclusively breastfed (in ‘months’, ‘years’, or ‘don't know’).

#### 2.6.3 Breastfeeding

All infant feeding survey items were adapted from local, state, and national infant feeding surveys [19, 20] and an outline of the definitions of breastfeeding is provided in Table 1.

- Ever breastfed (initiated)

**TABLE 1:** World Health Organization definitions of breastfeeding [1].

Feeding practice	Definition
Exclusive breastfeeding	Infant only receives breast milk including expressed milk, with the exception of medication (vitamins, minerals and oral rehydration solutions). Infant does not receive any solid or semi‐solid food or fluid including infant formula, water or non‐human milk.
Predominant breastfeeding	Infant receives breastmilk as the predominant source of nourishment. Infant may have the addition of water and water‐based drinks such as sweetened water, cordial, fruit drinks and soft drinks. Infant does not receive non‐human milk or food‐based fluids.
Any breastfeeding	Infant receives any breastmilk, whether exclusive or complementary (i.e., infant formula).
Ever breastfed	Infant has received breastmilk at least once.

In line with ANIFS, women were asked the following: ‘*Once your baby was born, did you try to breastfeed or give your baby breastmilk?*’. A woman was considered to have initiated breastfeeding if they had reported ‘yes’ to this item.

ii Breastfeeding status (any, predominant, exclusive)

As per the ANIFS, women were asked to report if their *‘baby had received breastmilk since this time yesterday*’. All women who reported ‘yes’ were deemed to be currently breastfeeding (any status). For predominant and exclusive breastfeeding, women were also asked if they had introduced formula; water; cow's milk and/or alternative; cordial; fruit juice; any fluid; and any form of soft/semi‐solid/solid foods (yes, no, don't know). As outlined in Table 1 exclusive breastfeeding was defined as a woman who was currently breastfeeding (yes) and had not introduced formula, water, cow's milk and/or alternative, cordial, fruit juice, any fluid, or any form of soft/semi‐solid/solid foods (responded ‘no’ to all). Predominant breastfeeding was defined as an infant who received breastmilk as the predominant source of nourishment. In addition, the infant may also have water and water‐based drinks.

iii Breastfeeding duration

Breastfeeding duration was assessed using ANIFS survey items. In accordance with Centre for Disease Control definitions of breastfeeding duration [21], women who had ceased breastfeeding were asked to report the age (in months) at which breastfeeding ceased.

iv Exclusive breastfeeding duration (age at which infant was first fed alternate foods/fluid)

Women were asked to report the age (months) the infant was first introduced to foods or fluids, using survey items from the ANIFS. Exclusive breastfeeding duration was calculated from respondents whose first feed was breast milk and the lowest reported age (months) at which food or other fluids, including formula, were introduced.

#### 2.6.4 Other Infant Feeding Practices

- Infant formula and other fluids

Women were asked to report if the infant had been fed formula in the last 24 h, using survey items from the ANIFS as follows: ‘*Since this time yesterday, has your baby drunk any infant formula products?*’. Women were considered to have introduced formula if they had reported ‘yes’ to this item. Of those that had introduced formula, women were then asked to report the age (in months) at which formula was first introduced to their infant. They were also asked ‘*Since this time yesterday, has your baby drunk any other fluids*’. Mothers were provided a list of fluids and asked if they baby had received the fluid since this time yesterday. If a mother responded ‘yes’ there were asked to report how old their baby was when they first had this drink. The list of fluids related to: (i) Plain Water; (ii) Sweetened or flavoured water; (iii) Cow's milk; (iv) Cordial; (v) Flavoured milk; (vi) 100% Fruit juice; (vii) Fruit drink; (viii) Non‐caffeinated soft‐drinks; and (ix) Caffeinated soft‐drinks.

ii Solids

Based on ANIFS items, women were asked to report if the infant had been fed solids as follows: ‘*Has your baby eaten any soft or semi‐solid or solid food?*’. A woman was considered to have introduced solids if they had reported ‘yes’ to this item. Of those who had introduced solids, women were asked to report the age of (a) first introduction (months) and (b) ‘regular’ introduction of solid foods (months). Of the women that had introduced solids, they are also asked ‘*What type of soft, semi‐solid or solid food has your baby had*’, whereby multiple response options could be selected including: (i) Dairy (milk, yoghurt, cheese, custard); (ii) Meat and proteins (beef, lamb, fish, eggs); (iii) Grains and cereals (rice cereal, oats, bread, pasta); (iv) Starchy vegetables (potato, sweet potato, tubers); (v) Legumes and/or nuts; (vi) Other Vegetables; (vii) Fruit; (viii) Candies, chocolate and other sugar confections; (ix) Frozen treats (ice cream, gelato, sorbet, popsicles); (x) Cakes, pastries, sweet biscuit; and (xi) Chips, crisps, fries, fried dough, instant noddles.

#### 2.6.5 Sources of Information for Infant Feeding

Women were asked to report from a list of 12 response options (see Table 5) which source of information they used *‘most frequently’* and found *‘most helpful’* to make decisions about (i) breastfeeding and formula feeding and (ii) their baby starting solids (women were only able to select one response for each question).

## 3 Data Analysis

Statistical analyses were performed using SAS 9.3 software. Data regarding the characteristics of the sample are presented categorically. Maternal age and timing of return to work after birth were trichotomized where maternal age was categorised as ‘18–24 years’, ‘25–34 years’ and ‘≥ 35 years’ and women's timing of return to work after birth was categorised as ‘0–3 months’, ‘4–6 months’, and ‘> 6 months’. Condensed response categories were created for (i) Aboriginal or Torres Strait Islander Origin (‘Aboriginal or Torres Strait Islander or both’ or ‘Neither Aboriginal or Torres Strait Islander’); (ii) maternal education (‘high school or less’ or ‘tertiary education or more’) and; women's current employment status including ‘employed’ (full time, part time or casual); ‘maternity leave’ (paid or unpaid) or ‘unemployed’ (home duties, unemployed, retirees or full time carers). Women's residential postcode was used to determine socioeconomic area using the 2016 socioeconomic indexes for areas using the index of socio‐relative disadvantage [22]. Women's residential postcode was also used to determine geographical remoteness (‘major cities’ or ‘regional/remote’) using the Access/Remoteness Index of Australia [23]. Infants' weight for age percentile at birth was dichotomized into those that were above the 50th percentile and those that were at or below the 50th percentile of the CDC growth charts [21]. Knowledge of exclusive breastfeeding recommendations was dichotomized into two categories (i) those that answered ‘6 months’ and (ii) all other responses.

Descriptive statistics were used to detail the mother and infant demographics, as well as some breastfeeding and other infant feeding practices (average duration of breastfeeding, timing of introduction to solids (first introduction; regular introduction), age of formula introduction, % that initiated breastfeeding). Due to the non‐normal distribution of the data, the median was used to examine the duration of breastfeeding and age of formula introduction. However, when the data was normally distributed, the mean was used.

When looking at events such as breastfeeding (any, predominant and exclusive) and other infant feeding practices (introduction to solid foods and formula) some participants had not yet experienced the event at the time of the survey. As we are unable to predict when the event (e.g., breastfeeding cessation) will occur in the future, these responses were censored. For example, for exclusive breastfeeding, if a participant had not yet experienced the disqualifying event at the time of the survey (e.g., stopped exclusively breastfeeding) their response was censored. A Kaplan–Meier survival analysis was used to estimate the cumulative proportion surviving the event and associated 95% confidence intervals. This method is consistent with international practice on reporting breastfeeding [24]. As the introduction to solids and infant formula was about having an attribute (e.g., introduced solids) rather than experiencing a disqualifying event (i.e., stopping) and rates increase over time (with the age of the child) [10] a ‘reverse’ survival analysis was produced where we estimated one minus the cumulative proportion surviving at each event [25].

## 4 Results

### 4.1 Characteristics of Sample

Of the 713 women that were approached, a total of 356 eligible mothers consented to participate in the study exploring infant feeding practices (50% response rate). As displayed in Table 2, the majority of respondents were aged between 25 and 34 years (68%), had infants aged 7 months at the time of the survey (43%), did not identify as Aboriginal and/or Torres Strait Islander (93%), were tertiary educated (71%), resided in areas of socioeconomic disadvantage (62%) and in major cities within the HNELHD (57%). The majority of women (65%) had correct knowledge of the length of time to exclusively breastfeed for, and approximately half of the infants were above the 50th weight‐for‐age percentile at birth.

**TABLE 2:** Demographic characteristics of mother and infant.

Sociodemographic characteristics	*n* ^a^ (%)
All	356 (100)
Age of mother (years)	
18–24	45 (13)
25–34	242 (68)
35+	69 (19)
Age of infant (months)	
6	133 (37)
7	154 (43)
8	69 (19)
Mother identified as aboriginal and/or Torres Strait Islander	
Yes	23 (7)
No	325 (93)
Country of birth	
Australia	314 (88)
Other	42 (12)
Highest level of education	
High school or less	110 (29)
Tertiary	250 (71)
Current employment status	
Employed	140 (39)
Maternity leave (paid/unpaid)	140 (39)
Unemployed	76 (21)
Socioeconomic status^b^	
Most disadvantaged	222 (62)
Least disadvantaged	134 (38)
Level of remoteness^c^	
Major cities	202 (57)
Regional/remote	154 (43)
Infant weight^d^	
> 50th percentile	172 (50)
< 50th percentile	173 (50)
Knowledge of exclusive breastfeeding guidelines (6 months)	
Yes	232 (65)
No	124 (35)

^a^ Total number of participants across characteristics may not add up to the overall total number of participants due to missing or ineligible data.

^b^ Socioeconomic status was calculated using the 2016 socioeconomic indexes for areas (SEIFA).

^c^ Level of remoteness was determined using the Access/Remoteness Index of Australia.

^d^ Infant weight was dichotomized using the CDC weight for age percentiles.

### 4.2 Breastfeeding

Breastfeeding was initiated in 97% (*n* = 346) of infants. In their first month of life, 81% of infants were exclusively breastfed, with 51% exclusively breastfed to 4 months and 1% exclusively breastfed to 6 months of age (Table 3). Eighty‐nine percent of infants received ‘any’ breastmilk in the first month of life, with 69% being breastfed to 4 months and 64% to 6 months of age. Of those that had ceased breastfeeding at the time of the survey (*n* = 128/346, 37%), the median duration was 1.8 months (IQR: 0.7–4.0) for any breastfeeding status and 1.2 months (IQR 0.2–3.8) for exclusive breastfeeding.

**TABLE 3:** Cumulative proportion of children exclusively breastfed, predominantly breastfed, or receiving any breastmilk to each month of age using a Kaplan‐Meier survival analysis.

Age (to months)	Exclusively breastfed^a^ % (95% CI)	Predominantly breastfed^b^ % (95% CI)	Receiving any breastmilk^c^ % (95% CI)
0 to < 1	81 (77, 85)	81 (77, 85)	89 (86, 92)
1	74 (69, 78)	74 (69, 79)	81 (77, 85)
2	71 (66, 75)	71 (66, 76)	78 (73, 82)
3	66 (61, 71)	67 (62, 72)	74 (69, 78)
4	51 (46, 57)	52 (47, 57)	69 (64, 74)
5	27 (23, 32)	29 (24, 33)	67 (61, 71)
6	1 (0, 3)	2 (1, 3)	64 (58, 69)
7	1 (0, 3)	1 (0, 2)	63 (58, 68)
8	1 (0, 3)	1 (0, 2)	62 (56, 67)

^a^ Exclusive Breastfeeding: the infant is receiving only breast milk and no other liquids or solids.

^b^ Predominant Breastfeeding: the infant is receiving almost all of its nutrients from breast milk but takes some other liquids such as water, oral rehydration solutions, and drops or syrups.

^c^ Any Breastfeeding: infant is receiving some breast milk [2].

### 4.3 Other Infant Feeding Practices

- Infant formula feeding and intake of other fluids

As shown in Table 4, in the first month of life 21% of infants had been introduced to infant formula. At 4 months, 43% of infants were having infant formula and by 8 months this was 57%. Of those that had been introduced to formula, the median timing of introduction was at 1.5 months of age (IQR: 0.2–4).

**TABLE 4:** Cumulative proportion of children who were introduced to soft/semi‐solid/solid food and infant formula by each month of age using a Kaplan‐Meier survival analysis.

Age (to months)	Introduced solids % (95% CI)	Regularly eating solids % (95% CI)	Introduced to formula % (95% CI)
0 to < 1	0 (0, 1)	0 (0, 1)	21 (17, 26)
1	0 (0, 1)	0 (0, 1)	28 (24, 33)
2	0 (0, 1)	0 (0, 2)	31 (26, 36)
3	1 (0, 3)	1 (0, 2)	34 (30, 40)
4	22 (18, 27)	5 (3, 8)	43 (38, 48)
5	56 (51, 61)	25 (21, 30)	46 (41, 51)
6	98 (96, 99)	81 (76, 85)	51 (46, 56)
7	100 (98, 100)	96 (93, 98)	54 (49, 59)
8	100 (98, 100)	98 (96, 99)	57 (50, 63)

At the time of the survey, 69% (*n* = 130) of infants had consumed ‘other’ fluids in the previous 24 h. Approximately 88% had introduced water, and the mean age of water introduction was 5.8 months (SD: 1.14). Only a small proportion of mothers reported introducing ‘other’ fluids, most notably: 3% (*n* = 8) had introduced cow's milk; 2% (*n* = 6) had introduced 100% fruit juice; 2% (*n* = 6) had introduced fruit drink. No mother reported introducing caffeinated soft drinks.

ii Introduction to solids

Infants were first introduced to solids at 5.3 months (reported as the mean, SD 0.81). The mean age of regular introduction of solids was 5.9 months (SD 0.79). Just under one quarter (22%) of children were first introduced to solid foods at 4 months, 56% had been introduced to solid foods at 5 months and 98% at 6 months. At 6 months, 81% of children were regularly consuming solids and by 8 months this was 98% (See Table 4).

Table 5 presents the types of soft, semi‐solid, and solid foods that have been introduced to infants. Notably, 97% of mothers had introduced their infants to fruit, 96% had introduced a starchy vegetable, and 86% had introduced ‘other’ vegetables. Approximately 14% had also introduced cakes, pastries, and sweet biscuits; 13% had introduced frozen treats; and 9% had introduced fried chips and crisps.

**TABLE 5:** Type of soft, semi‐solid, or solid food introduced to infants (*n* = 349).

Food type	Introduced % (*n*)
Fruit	97 (340)
Starchy vegetables (potato, sweet potato, other tubers)	96 (336)
Other vegetables	86 (299)
Grains and cereals (rice cereal, oats, bread, pasta)	85 (297)
Meat and proteins (beef, lamb, fish, eggs)	75 (262)
Dairy (milk, yoghurt, cheese, custard)	65 (226)
Legumes and/or nuts	40 (141)
Cakes, pastries, sweet biscuits etc.	14 (50)
Frozen treats (ice cream, gelato etc.)	13 (44)
Chips, crisps, fries and similar items	9 (31)
Candies, chocolate and other confections	5 (19)

### 4.4 Sources of Information for Infant Feeding

As shown in Table 6 the ‘most frequently’ used source of information for breastfeeding or formula feeding included friends and family (30%), child and family health nurses (15%), websites (12%) and social media/blogs (11%). The ‘most helpful’ source of information to make decisions about breastfeeding or formula feeding were friends and family (23%), child and family health nurses (23%), ‘other health professionals’ (e.g., lactation consultants) (13%) and websites (8%). The ‘most frequently’ used source of information for starting solids included friends and family (33%), social media/blogs (18%), websites (11%) and ‘other’ (10%). The ‘most helpful’ source of information for starting solids were friends and family (28%), social media/blogs (16%), child and family health nurses (15%) and ‘other’ (9%). Within the ‘other’ category women commonly specified ‘books’ as ‘frequently used’ and ‘helpful’ sources of information for starting solids.

**TABLE 6:** The sources of information used *‘most frequently’* and identified as *‘most helpful’* to make decisions about breastfeeding or formula feeding and starting solids.

	Breastfeeding or formula feeding^a^				Starting solids^a^			
	Most frequent		Most helpful		Most frequent		Most helpful	
Source of information	*n* (%)	Rank	*n* (%)	Rank	*n* (%)	Rank	*n* (%)	Rank
Friends and family	96 (30)	1	73 (23)	2	100 (33)	1	86 (28)	1
Child and family health nurse	48 (15)	2	74 (23)	1	29 (9)	5	45 (15)	3
Websites	40 (12)	3	26 (8)	4	33 (11)	3	27 (9)	5
Social media/blog	37 (11)	4	25 (8)	6	54 (18)	2	50 (16)	2
Other health professional^b^	27 (8)	5	41 (13)	3	8 (3)	9	17 (6)	7
General practitioner	22 (7)	6	22 (7)	7	14 (5)	7	23 (7)	6
Other^b^	19 (6)	7	26 (8)	5	32 (10)	4	28 (9)	4
The blue book	7 (2)	9	3 (1)	11	0 (0)	11	5 (2)	10
Apps	5 (2)	10	1 (< 1)	12	18 (6)	6	14 (5)	8
Pharmacy nurse	3 (1)	11	7 (2)	10	7 (2)	10	6 (2)	9
Telephone helpline	2 (1)	12	11 (3)	9	0 (0)	11	0 (0)	12
Refused/Don't know	16 (5)	8	12 (4)	8	10 (3)	8	3 (1)	11

*Note:* World Health Organisation. Infant Feeding Guidelines: Summary, 2013 [02/05/2022]. Available from: https://www.who.int/health‐topics/breastfeeding#tab=tab_1. National Health and Medical Research Council. Literature Review: Infant Feeding Guidelines. Canberra; 2012.

^a^ Women were only able to select one ‘source of information’ option across each question. Due to missing data the total number of participants for the breastfeeding (*n* = 324) and solids questions (*n* = 307) do not add up to the total number of participants in sample (*n* = 356).

^b^ When participants selected this response option, they were asked to ‘specify’ the source of information used.

## 5 Discussion

This study provides valuable insight into the prevalence of breastfeeding and early infant feeding practices among infants from birth to 8 months in the HNE region of New South Wales, Australia. Broadly aligned with decade‐old results from the ANIFS (2010) and the more recent OzFITS study (2021), these findings indicate that while breastfeeding initiation rates by mothers were high (97%), only 64% of infants were still receiving any form of breastmilk to 6 months of age. Furthermore, of those who had ceased breastfeeding, the average duration of breastfeeding (any status) and exclusive breastfeeding was 1.8 and 1.2 months, respectively. These results highlight that more must be done within the HNE region to support mothers to sustain exclusive breastfeeding practices beyond the first few months, as well as to continue breastfeeding to 6 months and beyond as a key preventive health priority to support healthy infant growth and development.

Despite the data showing high breastfeeding initiation rates, only 1% of infants in HNE were exclusively breastfed at 6 months, consistent with the 1% and 2% found in the OzFits and ANIFs studies respectively [19, 25]. In contrast to previous research, HNE infants had much lower rates of formula introduction in the first month (21%) compared to both the OzFITS and ANIFs studies (~40% respectively). By 6 months, however, formula feeding was broadly consistent, with 51% of infants in HNE receiving infant formula compared to 56% (OzFITS) and 55% (ANIFs) respectively [19, 25]. The initial discrepancy in formula feeding (within the first month) may be due, in part, to an additional survey question included in both national surveys that specifically asked women about formula feeding prior to discharge from hospital. Another inconsistent finding was that HNE had a much lower breastfeeding duration (1.8 months; any breastmilk) than that reported in the OzFITS study of 11 months (IQR 10.2–11.8) [25]. Again, this is likely to reflect differences in the study methodology. For example, the age of the study sample included infants aged 6–8 months compared to the OzFITS sample that ranged from birth up to 2 years.

Current recommendations state that ‘*at around 6 months, it is recommended iron‐rich nutrient dense foods are introduced to complement breast milk* [1], *with continuation of breastfeeding to 12 months and beyond*’ [2]. While our analysis found the mean age of solids introduction was 5.3 months, which is broadly consistent with recommendations and other Australian‐based findings [25], 22% of HNE infants were introduced to solids before 4 months. This result is not desirable from a public health perspective, as large population‐based cohort studies have found that early introduction to solids (at or before 4 months) is associated with an increased risk of childhood overweight [26, 27]. For example, a large population‐based cohort of Australian 1‐year‐old infants (*n* = 3153) found that infants introduced to solids at or before 4 months were approximately 3 times more likely to have an above‐normal body mass index compared with those introduced to solids at 6 months [27]. As such, our data suggest a need to not only support mothers in sustaining breastfeeding during the first 6 months, but also ensure the timing of solids introduction is better aligned with recommendations.

Our study found that 88% of infants had been introduced to water by 6–8 months, and a low proportion of infants had been introduced to other fluids, including cow's milk (3%), 100% fruit juice (2%) and fruit drink (2%). While these behaviours are broadly aligned with the Australian infant feeding guidelines for other fluids [2], the introduction of these fluids before 6 months interferes with intakes of breastmilk or infant formula, indicating that a small proportion of parents may require guidance on delaying the introduction of these other fluids. When it comes to introducing solids, the Australian infant feeding guidelines suggest ‘*the introduction of solid foods at around 6 months should start with iron‐containing foods, including iron‐enriched infant cereals, pureed meat, poultry and fish (all sources of haem iron), or cooked tofu and legumes. Vegetables, fruits, and dairy products such as full‐fat yoghurt, cheese and custard can than be added’* [2]. Results from our study indicate that current practices are not consistent with this recommendation as only 75% of infants had been introduced to meats and proteins (e.g., eggs), and only 40% had been introduced to legumes and/or nuts, compared with 97% that had been introduced to fruit (a low iron food), and 96% had been introduced to starchy vegetables. In addition, infant feeding guidelines state ‘*the consumption of nutrient‐poor foods with high levels of fat/saturated fat, sugar, and/or salt (e.g. cakes, biscuits, confectionary and potato chips) should be avoided or limited’* [2], yet, alarmingly the introduction of cakes and biscuits (14%), frozen treats like ice‐cream (13%) and fried chips and crisps (9%) were relatively prevalent too. Suggesting that parents may require additional education/support to introduce appropriate and nutrition supporting first foods to their infants.

This study found that women used a variety of sources of information to make decisions about infant feeding, with ‘friends and family’, ‘child and family health nurses’ and digital sources (websites, social media) being frequently used and perceived as helpful for making decisions about breastfeeding, formula feeding and starting solids. These findings are consistent with international literature [28, 29, 30]. For example, a cross sectional survey undertaken with 561 Canadian mothers of infants (aged 6 months) found that the internet/websites (84%–93%), family (79%), friends (78%) and health professionals (> 50%) were the highest used and valued sources of information related to infant feeding, sleep and infant growth and development [28]. While the attitudes and beliefs of friends and family about breastfeeding and formula feeding are major influences on infant feeding choices [11, 31, 32], they are not always consistent with recommended guidelines [33]. For example, a qualitative evidence synthesis that explored parent's barriers and experiences to meeting infant feeding guidelines found that family and friends were common sources of advice for infant feeding, however 10 of the 13 included studies reported that advice received was ‘outdated’ or inconsistent with infant feeding guidelines [29].

Digital technologies/resources offer opportunities to support the delivery of evidence‐based infant feeding information in real time [34]. However, such information can be difficult to locate, hard to understand, and is not always credible or reliable [35, 36]. A recent systematic review, including 16 studies, found that combining educational activities with web‐based personalised support through discussion forums appeared to be the most effective way to improve breastfeeding outcomes and long‐term exclusive breastfeeding rates [37]. As such, the delivery of clear, consistent, and evidence‐based information from credible sources via a range of modalities may present an opportunity to support families to meet evidence‐based infant feeding guidelines. Furthermore, given maternal reliance on family and friends for infant feeding advice, interventions that include strategies targeting a mother's support network (family and friends) may be warranted. This notion is supported by the literature, whereby a meta‐analysis including 43 studies found that antenatal and postnatal support provided to both mothers and family members at home increased exclusive breastfeeding up to 6 months by 48% (95% CI, 32%–66%) and continued breastfeeding for 12–23 months by 26% (95% CI, 5%–50%) [38].

The strengths of this study include its relatively large sample size and survival analysis techniques used to explore infant feeding practices consistent with international and national standards [19, 24]. We also used survey items consistent with previous National surveys on infant feeding practices to enable comparisons to be made. These results provide important insights into current infant feeding practices across a wide geographic and socio‐economic diverse region of Australia. However, as the study was undertaken in one geographical region of Australia, the results may not generalise to the broader population. Furthermore, the accuracy of recall at 6–8 months may not be a true representation of when breastfeeding ceased or formula or solids commenced; however, this dietary‐recall approach is consistent with existing population‐based surveys [19, 25].

In conclusion, this study reports on the feeding practices of infants aged 0–8 months, living within a geographically and socioeconomically diverse region of Australia, and explores what sources of information mothers frequently access to inform their infant feeding behaviours. Like other Australian studies more broadly, the findings demonstrate that exclusive breastfeeding for the first 6 months of life should remain a significant focus of public health nutrition efforts. Our results demonstrate a gap between the current infant feeding guidelines and maternal infant feeding practices, including the duration of exclusive breastfeeding, early introduction of solids (i.e., before 6 months) and selection of first foods to optimally support healthy infant growth and development. Given mothers make infant feeding decisions that are frequently influenced by their close support network (i.e., partner, family and friends), future breastfeeding initiatives should seek to target mothers and their support networks. Furthermore, the provision of consistent, evidence‐based, and trusted information via a range of modalities at key infant feeding milestones is recommended to further support public health messaging around infant feeding practices.

## Author Contributions

R.S. obtained funding; T.D. and N.H. conceived the research question; R.S., L.W., J.W., P.C., M.H., S.R., D.G., N.H. and T.D. were part of the advisory group that advised on the background and study methodology; T.D. and S.Y. lead the data collection; C.L. lead the data analysis; T.D. and J.J. drafted the manuscript. All authors have read and agreed to the published manuscript.

## Ethics Statement

The study was conducted according to the guidelines of the Declaration of Helsinki and approved by Hunter New England Human Research Ethics Committee (16/11/16/4.07), Aboriginal Health and Medical Research Council (1236/16) and the University of Newcastle Human Research Ethics Committee (H‐2017‐0032).

## Consent

Informed consent was obtained from the participants prior to data collection.

## Conflicts of Interest

The authors declare no conflicts of interest.

## Data Availability

The data that support the findings of this study are available on request from the corresponding author. The data are not publicly available due to privacy or ethical restrictions.
